# Virucidal Coatings Active Against SARS-CoV-2

**DOI:** 10.3390/molecules29204961

**Published:** 2024-10-20

**Authors:** Anna I. Barabanova, Eduard V. Karamov, Viktor F. Larichev, Galina V. Kornilaeva, Irina T. Fedyakina, Ali S. Turgiev, Alexander V. Naumkin, Boris V. Lokshin, Andrey V. Shibaev, Igor I. Potemkin, Olga E. Philippova

**Affiliations:** 1 Institute of Organoelement Compounds, 119991 Moscow, Russia; naumkin@ineos.ac.ru (A.V.N.); bloksh@ineos.ac.ru (B.V.L.); 2 Gamaleya National Research Center for Epidemiology and Microbiology of the Russian Ministry of Health, 123098 Moscow, Russia; karamov2004@yandex.ru (E.V.K.); vlaritchev@mail.ru (V.F.L.); kornilaeva@yandex.ru (G.V.K.); irfed2@mail.ru (I.T.F.); turgiev@ld.ru (A.S.T.); 3 Physics Department, Moscow State University, 119991 Moscow, Russia; shibaev@polly.phys.msu.ru (A.V.S.); phil@polly.phys.msu.ru (O.E.P.); 4 Chemistry Department, Karaganda E.A. Buketov University, Karaganda 100028, Kazakhstan

**Keywords:** SARS-CoV-2, COVID-19, cationic surfactants, virus inactivation, quaternary ammonium compounds, disinfectants

## Abstract

Three types of coatings (contact-based, release-based, and combined coatings with both contact-based and release-based actions) were prepared and tested for the ability to inactivate SARS-CoV-2. In these coatings, quaternary ammonium surfactants were used as active agents since quaternary ammonium compounds are some of the most commonly used disinfectants. To provide contact-based action, the glass and silicon surfaces with covalently attached quaternary ammonium cationic surfactant were prepared using a dimethyloctadecyl[3-(trimethoxysilyl)propyl]ammonium chloride modifier. Surface modification was confirmed by attenuated total reflection infrared spectroscopy, X-ray photoelectron spectroscopy (XPS), scanning electron microscopy, and contact angle measurements. The grafting density of the modifier was estimated by XPS and elemental analysis. To provide release-based action, the widely used quaternary ammonium cationic disinfectant, benzalkonium chloride (BAC), and a newly synthesized cationic gemini surfactant, C18-4-C18, were bound non-covalently to the surface either through hydrophobic or electrostatic interactions. Virus titration revealed that the surfaces with combined contact-based and release-based action and the surfaces with only release-based action completely inactivate SARS-CoV-2. Coatings containing only covalently bound disinfectant are much less effective; they only provide up to 1.25 log10 reduction in the virus titer, probably because of the low disinfectant content in the surface monolayer. No pronounced differences in the activity between the flat and structured surfaces were observed for any of the coatings under study. Comparative studies of free and electrostatically bound disinfectants show that binding to the surface of nanoparticles diminishes the activity. These data indicate that SARS-CoV-2 is more sensitive to the free disinfectants.

## 1. Introduction

The coronavirus disease (COVID-19) caused by severe acute respiratory syndrome coronavirus 2 (SARS-CoV-2) was declared a public health emergency of international concern [1]. COVID-19 has quickly spread throughout the world, and on 11 March 2020, it was recognized as a global pandemic by the World Health Organization [2,3]. With the rapid mutations of SARS-CoV-2 accompanied by changes in its features and contagions, the fighting against this disease is quite challenging. Nowadays, no specific therapy that can address all variants and mutants of this virus at once is available [4]. Regarding the vaccines, they should be constantly adapted to emerging mutations of SARS-CoV-2, which requires considerable time [4]. Under those conditions, effective inactivation of the virus becomes quite important to control the spreading of the infection.

It was agreed that the spread of SARS-CoV-2 occurs predominantly through direct human-to-human transmission or via intermediate fomites, aerosols, or droplets sent into the air when an infected person coughs, sneezes, or talks [5]. At the same time, contact transmission also plays an important role, since infected persons can touch surfaces (door handles, light switches, etc.) or their aerosol droplets might settle on surfaces, leading to their contamination [6]. It was shown that at ambient conditions the virus can remain viable and infectious for 3–4 days on plastics and stainless steel [7,8,9], for up to 2 days on glass and banknotes [8], and for up to 24 h on cardboard [9]. When somebody touches a contaminated surface and then touches his eyes, nose, or mouth, the pathogens penetrate the body [5] (note that humans can touch their eyes, nose, and mouth up to 200 times a day [10]). Given the significance of surface contamination in the spread of the virus, special attention should be paid to the elaboration of surface coatings that can effectively inactivate SARS-CoV-2 [11].

The simplest way to eliminate the coronavirus from surfaces is their treatment with disinfectants. The list of COVID-19 disinfectants provided by the Environmental Protection Agency [12] includes quaternary ammonium compounds (mainly cationic surfactants), peroxides, alcohols, phenols, etc. Those disinfectants efficiently inactivate SARS-CoV-2 [13,14,15,16,17,18,19], but their antiseptic action does not last long because they are easily washed away [20]. To provide long-term effectiveness, they should be reused frequently, which can cause undesirable toxicological effects [4] including the risk of respiratory health outcomes, e.g., asthma [21], and pollution of the environment [22,23,24].

To minimize the toxic effects, surfaces with prolonged activity against the virus are needed. They can be produced by linking an active substance to the surface. Depending on the mode of action, the surfaces with long-lasting activity can be contact-based or release-based [25]. In contact-based systems, the active agent is covalently attached, whereas in release-based systems it is linked non-covalently to the surface (e.g., bound electrostatically). In the latter case, the agent exhausts over time, thereby reducing the lifetime of the activity. Covalently attached agents are strongly bound to the surface and do not leach out, but their activity may be lower in comparison with free agents [26]. To solve the problem of the balance between the activity and the duration of the action, one can use systems containing both covalently and non-covalently linked agents. For instance, Druvari et al. [26] elaborated coatings based on poly(4-vinylbenzyl chloride-co-acrylic acid) and poly(sodium 4-styrenesulfonate-co-glycidyl methacrylate) copolymers modified by covalent and/or electrostatic binding of biocides (4-vinyl benzyl dimethylhexadecylammonium chloride and cetyltrimethylammonium 4-styrenesulfonate, respectively). It was observed [26] that the system with the electrostatically bound biocide is more active against *S. aureus* and *P. aeruginosa*. But the most promising results were obtained for the coatings containing both covalently and non-covalently attached biocides [26].

By now, only a few biocidal surfaces have been tested for activity against SARS-CoV-2. They represent mainly release-based systems composed of negatively charged polymers with electrostatically bound cationic surfactants acting as disinfectants. Those surfactants are known to inactivate various bacteria and enveloped viruses, including SARS-CoV-2, by disrupting their lipid bilayer envelopes [18,27]. In a recent report [28], antiseptic polymer gel–surfactant complexes were prepared by incorporating the cationic disinfectant cetylpyridinium chloride into the oppositely charged, slightly cross-linked matrices of copolymers of acrylamide and sodium methacrylate (or sodium 2-acrylamido-2-methylpropane sulfonate) or copolymers of vinylpyrrolidone and sodium methacrylate. Also, a water-insoluble complex of polystyrene sulfonate with disinfectant benzalkonium chloride (BAC), exhibiting anti-SARS-CoV-2 activity, was elaborated [29]. However, to the best of our knowledge, no anti-SARS-CoV-2 coatings with covalently attached virucidal agents or coatings combining covalently and non-covalently attached virucidal agents have been proposed thus far.

The microstructure of surfaces may also play an important role in their activity since it also contributes to the way the surface interacts with viruses. For instance, hydrophobic and structured surfaces prevent the attachment of viruses. Balancing a strategy to repel attachment while delivering reactive chemical biocidal efficacy is a well-known conundrum investigated to counter surface fouling [30,31,32]. However, no studies on the effect of the roughness of surfaces containing both covalently and non-covalently attached biocides on their anti-SARS-CoV-2 activity have been performed.

**Figure 1:**
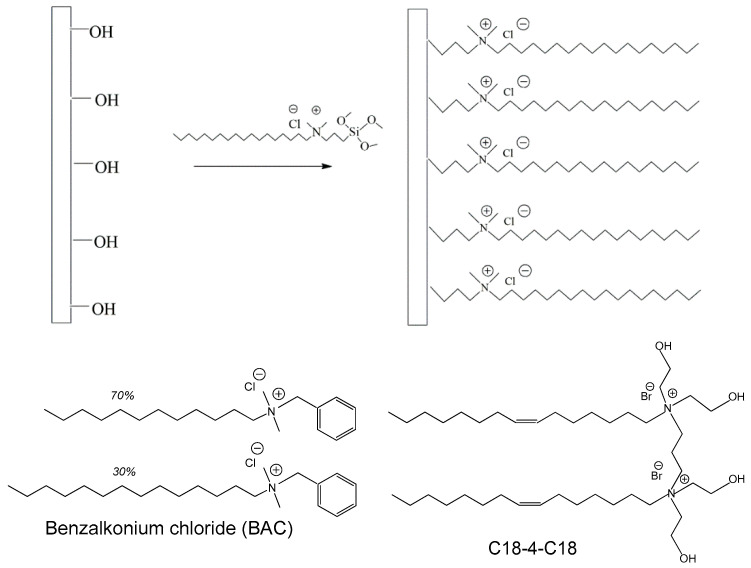
Schematic representation of the functionalization of an OH-group-containing surface with dimethyloctadecyl[3-(trimethoxysilyl)propyl]ammonium chloride and the chemical structure of cationic surfactants benzalkonium chloride (BAC) and N1,N1,N4,N4-tetrakis(2-hydroxyethyl)-N1,N4-di((Z)-octadec-9-enyl)butane-1,4-diaminium bromide (C18-4-C18) used for non-covalent surface modification.

The aim of the present work is to investigate surfaces that exhibit activity against SARS-CoV-2 based on combined contact-based and release-based action and to compare the activity of flat and structured surfaces. To provide contact-based antiviral action, the surfaces were covalently modified with the silane functionalization agent dimethyloctadecyl [3-(trimethoxysilyl)propyl]ammonium chloride (DTSAC), which contains a cationic group coupled with a long C18 hydrophobic tail (Figure 1) like that found in many quaternary ammonium disinfectants. To provide release-based antiviral action, one of the most common quaternary ammonium disinfectants, BAC, was bound non-covalently to the surface. In addition, a newly synthesized cationic gemini surfactant N1,N1,N4,N4-tetrakis(2-hydroxyethyl)-N1,N4-di((Z)-octadec-9-enyl)butane-1,4-diaminium bromide (C18-4-C18) [33] was tested instead of BAC. To provide roughness, the surface was covered by elongated nanoparticles (NPs) modified in the same way as the flat surface. It was demonstrated that coatings combining covalently and non-covalently bound surfactants are much more effective in fighting against SARS-CoV-2 than those containing only covalently bound surfactants. No pronounced differences in the antiviral activity between the flat and structured surfaces were observed.

## 2. Results and Discussion

### 2.1. Modification of Surfaces

**Figure 2:**
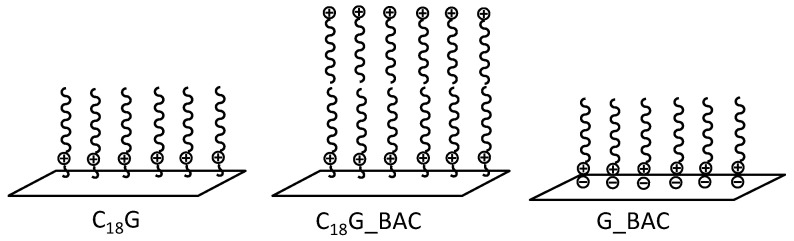
Schematic representation of a glass surface with covalently attached surfactant C_18_G, a glass surface combining covalently and non-covalently attached surfactants C_18_G_BAC, and a glass surface with non-covalently attached surfactant G_BAC.

DTSAC was covalently grafted onto the surface of glass platelets by a reaction between the surface silanol groups and methoxy groups of the modifier. A similar modification was also performed with the surface of the silicon Si wafer, which was covered by a thin SiO_2_ layer containing silanol groups [34]. The samples obtained upon the covalent attachment of DTSAC were denoted as C_18_G (Figure 2) and C_18_Si for glass G and silicon Si surfaces, respectively (Table 1).

**Figure 3:**
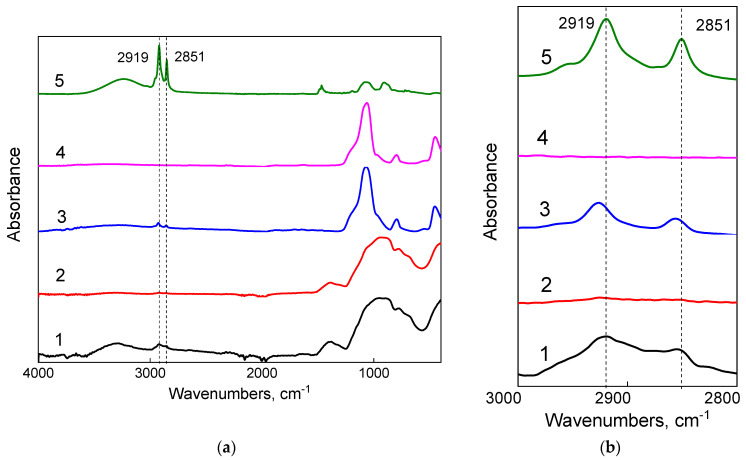
(**a**) ATR-IR-spectra of the modified glass substrate C_18_G (1) and NPs (3); the unmodified (initial) glass substrate (2) and unmodified NPs (4); and the modifier DTSAC (5). (**b**) Enlarged representation of ATR-IR-spectra in the range of C-H stretching vibrations for the modified C_18_Si (1) and NPs (3); the unmodified (initial) Si substrate (2) and unmodified NPs (4); and the modifier DTSAC (5).

The modification of the surfaces was confirmed by attenuated total reflectance infrared spectroscopy (ATR-IR) and X-ray photoelectron spectroscopy (XPS) (Table 2). In Figure 3, one can see that the ATR-IR-spectra of the modified surfaces (1) compared with the initial surfaces (2) demonstrate C−H stretching vibrations (2919 and 2851 cm^−1^) [32] characteristic of the alkyl groups of the modifier DTSAC (5), indicating successful modification.

**Figure 4:**
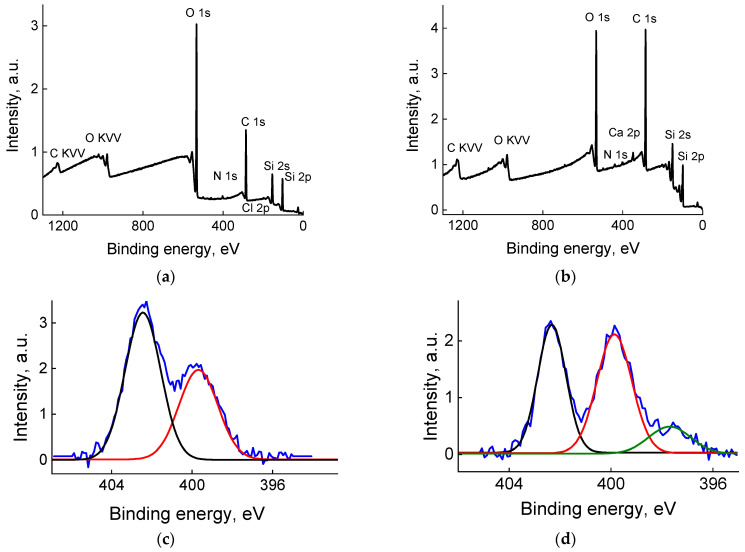
The survey XPS spectra of the C_18_G (**a**) and C_18_Si (**b**) samples and the high-resolution XPS spectra of the C_18_G (**c**) and C_18_Si (**d**) samples. Components attributed to different species are shown in different colors.

The XPS data (Table 2 and Table 3, Figure 4) for the modified surfaces revealed the presence of carbon and nitrogen atoms, which confirms the modification. Analysis of high-resolution XPS data showed that the most reliable information about modifier grafting can be obtained from N 1s spectra. They are presented in Figure 4c,d for samples C_18_G and C_18_Si. To determine the chemical state of nitrogen atoms, a curve-fitting procedure was carried out using Gaussian profiles and reliable chemical shifts [35]. The fitting results are listed in Table 3. The binding energies of 402.5 and 402.3 eV (Figure 4c,d, Table 3), characteristic of the (-CH_2_)_2_N^+^(CH_3_)_2_ groups, indicate the presence of a modifier on the substrate, while the binding energies of 399.7 and 399.9 eV (Figure 4c,d, Table 3) indicate the formation of bonds between the modifier and the substrate. The binding energy of 398.7 eV observed only for the C_18_Si sample (Figure 4d, Table 3) is due to the contamination of the substrate.

The atomic percentages of elements from the XPS data are summarized in Table 2. Note that this method analyzes the surface up to 10 nm in depth [36] and may include, in addition to the surface itself, some underlying layers. In the case of the silicon surface, XPS data allow for estimating the grafting density β from the atomic content of nitrogen N_N_ and oxygen N_O_ as β = 3N_N_/N_O_ × 6.8, taking into account that the modifier contains three oxygen atoms per one nitrogen (Figure 1), all oxygen atoms are located on the surface, and the density of Si atoms on a (100) plane is 6.8 atoms/nm^2^ [37]. In this way, it was determined that the grafting density of C_18_Si is equal to ca. 1 C_18_ group/nm^2^. From this value, one can estimate the packing density of the modifier λ_0_ as the ratio of the cross-sectional area of the surfactant chains A_CS_ to the available surface area per modifier on the substrate β. By setting the A_CS_ value (the van-der-Waals cross-sectional area of a surfactant chain) equal to 0.188 nm^2^ for an all-anti configured alkyl chain [38], one can obtain the packing density of the modifier on the silicon substrate as λ_0_ ≈ 0.2, which is rather low, suggesting that the alkyl chains are oriented mainly parallel to the surface and disordered [38].

**Figure 5:**
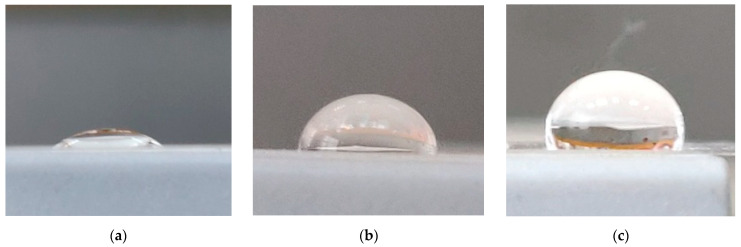
Photographs of original (**a**) and modified glass substrates (**b**,**c**) with flat C_18_G (**b**) and structured coatings C_18_G_NP (**c**).

The attachment of hydrophobic alkyl chains to the glass surface (Figure 2) as a result of the chemical modification enhanced the surface hydrophobicity, which was manifested as increased water contact angles. The static contact angles *θ*^H^_2_^O^ of the C_18_G and C_18_Si plates were found to be 75° and 85°, respectively, which were significantly higher than the contact angles of the original glass (28°) and Si (45°) platelets (Figure 5).

To create a structured surface, the C_18_G substrates were spin-coated with silica SiO_2_ NPs with covalently attached DTSAC. The DTSAC-modified silica NPs were synthesized in the same way as the chemically modified glass platelets. The attachment of DTSAC to the surface of the NPs was confirmed by ATR-IR spectroscopy, demonstrating the appearance of the bands at 2919 and 2851 cm^−1^ corresponding to the C−H stretching vibration of the methyl (or methylene) groups of the modifier (Figure 3). According to elemental analysis data (Table 2), the modified NPs contain 0.25 mmol DTSAC/g, which is twofold lower than the concentration of surface SiOH determined by titration (0.5 mmol/g [39]) and indicates that the surface contains one DTSAC molecule per two silanol groups. This corresponds to a grafting density β of 2.3 groups/nm^2^ taking into account that the concentration of the surface silanol groups per unit surface area in amorphous silica is 4.6 groups/nm^2^ [40]. This means that in the modified NPs, the footprint of one modifier is 0.4 nm^2^, which is comparable to the surface area per one surfactant molecule at full monolayer surface coverage of about 0.2 nm^2^ [38]. Therefore, the grafting density is rather high. Adding nanoroughness to the C_18_G substrates increased the static contact angle up to 98° (Figure 5), i.e., it made the surface more hydrophobic. Increased hydrophobicity of nanostructured surfaces was previously demonstrated in several studies [32,41,42,43,44]. It was attributed to the so-called Cassie–Baxter stable state [45], which is realized when air is entrapped between the liquid and solid phases, minimizing the contact area between them.

**Figure 6:**
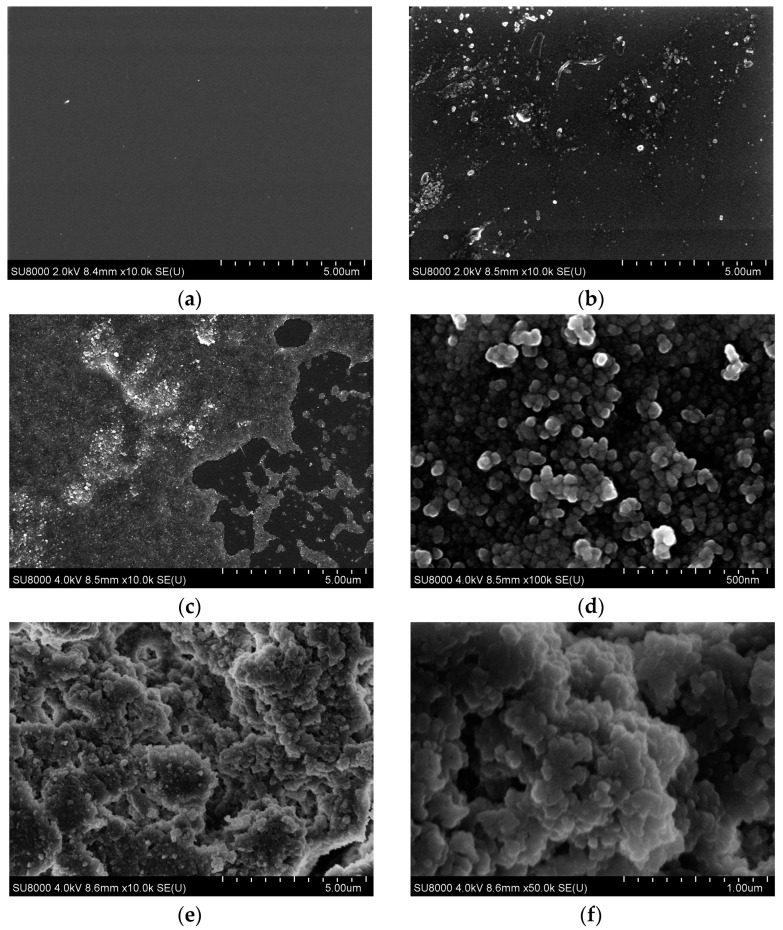
SEM images of the original substrate (**a**), covalently modified glass substrate C_18_G (**b**), and glass substrate covered with covalently modified silica NPs C_18_G_NP with 1 layer (**c**,**d**) and with 5 layers (**e**,**f**).

The morphologies of the original and covalently modified flat and structured glass substrates were also studied by scanning electron microscopy (SEM). As shown in Figure 6a,b, both the pure and DTSAC-modified glass substrates possess smooth surfaces. The deposition of only one layer of DTSAC-modified NPs led to the formation of a structured coating (Figure 6c,d), where individual elongated NPs with a diameter of 15–25 nm and a length of 48–100 nm can be observed (Figure 6d); their dimensions practically coincide with the size of the NPs in the initial suspension of NPs modified with DTSAC. However, at the large scale (Figure 6c), one can see some areas not covered with NPs (dark areas).

The application of five layers of 1 wt% suspension of SiO_2_ particles seems to contribute to a more complete coating of the glass surface, which is clearly seen in the large-scale SEM image (Figure 6e). On a smaller scale (Figure 6f), one can observe NPs that are self-assembled into microaggregates located on top of each other. The aggregates have a diameter of 60–70 nm and a length of 100–110 nm.

The coatings combining covalently and non-covalently attached surfactants C_18_G_BAC (Table 1) were obtained by pouring an aqueous BAC solution on the C_18_G samples, followed by air drying overnight. In the resulting coatings, BAC molecules were attached to the surface via hydrophobic interactions [46,47] with alkyl tails of covalently attached DTSAC (Figure 2).

To obtain samples with only non-covalently attached surfactant G_BAC or G_C18-4-C18 (Table 1), the negatively charged glass substrates (prepared by treating the initial glass substrates with 0.1 N NaOH) were coated with an aqueous solution of BAC or C18-4-C18 and then left to dry in air at room temperature overnight. In the coatings thus prepared, surfactant molecules were attached to the surface via electrostatic interactions (Figure 2).

Thus, three series of surface-functionalized platelets differing in the type of binding of the cationic surfactant (covalent, non-covalent, and combined covalent/non-covalent), chemical composition (DTSAC, BAC, C18-4-C18), and the flatness of the coating (flat and structured) were prepared.

### 2.2. Virus Inactivation

**Figure 7:**
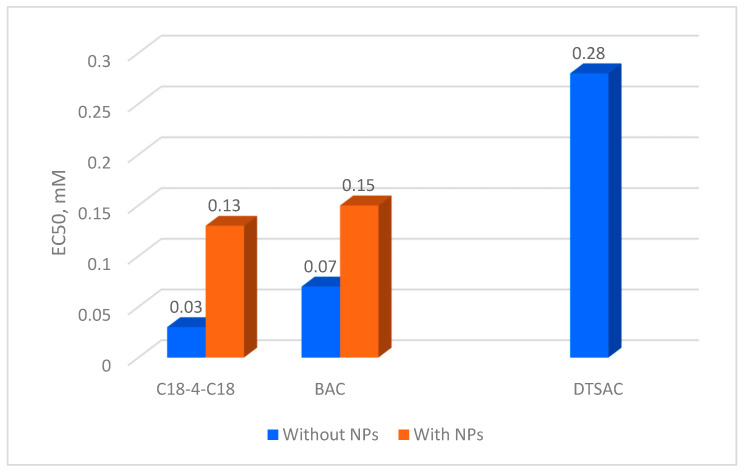
SARS-CoV-2 inactivation by free surfactants C18-4-C18, BAC, and DTSAC and surfactants electrostatically bound to negatively charged silica nanoparticles NP- at a constant surfactant/NP- mass ratio q. q = 0.6 for C18-4-C18, q = 0.17 for BAC (incubation time of 60 min).

First, the activity of three cationic surfactants under study against SARS-CoV-2 was investigated, and their 50% effective concentrations (EC50s) were determined (Figure 7). It was shown that the newly synthesized gemini surfactant C18-4-C18 is the most effective among them. Its EC50 value (0.03 mM) is 2.3-fold lower than that of the widely used disinfectant, cationic surfactant BAC. This is consistent with the recent data [33] demonstrating that gemini cationic surfactants have higher SARS-CoV-2-inactivating efficacy than one-tail surfactants.

The results of cytotoxicity tests for these compounds are presented in Table 4. A comparison with the EC50 values (Figure 7) shows that BAC has an EC50 of 0.07 mM at 1 h of incubation and a 50% cytotoxicity concentration (CC50) of 0.3 mM at a much longer incubation (72 h). The situation with other surfactants is similar. For DTSAC, EC50 is 0.28 mM vs. CC50 of 4.2 mM; for the gemini surfactant C18-4-C18, EC50 is 0.03 mM vs. CC50 of 0.8 mM. The considerable difference in incubation times between the experiments assessing the virus-inactivating efficacy of surfactants and their cytotoxicity (1 h vs. 72 h, respectively) indicates that incubating the cells with surfactants for 1 h (instead of 72 h) would probably require orders of magnitude higher concentrations to render adverse effects detectable. Thus, our study of SARS-CoV-2 inactivation was carried out well beyond the zone of toxic concentrations of surfactants. Note that adding NPs significantly increased the CC50 values, thereby decreasing surfactant cytotoxicity, which may be underlain by the surfactant binding to the NP surface and the resulting drop in the concentration of free surfactant in the solution.

Testing of the surfaces was performed by immersing the platelets in the virus-containing liquid and measuring the reduction in the viral titer. The results of the tests are summarized in Table 5. Let us first consider the data for the flat surfaces. In Table 5, one can see that the coatings containing only the covalently bound disinfectant (C_18_Si and C_18_G) do not demonstrate pronounced activity, even after prolonged incubation with the virus-containing liquid (60 min): the inhibition coefficient (IC) is 20.8% for C_18_Si and only 5.2% for C_18_G. This may be due to the small amount of disinfectant bound to the surface. The same amount of non-covalently attached C18-4-C18 produces a similar small effect (G_C18-4-C18, Table 5).

To augment the efficiency, higher concentrations of disinfectants need to be used. This can be achieved by their non-covalent binding to the surface. In this case, instead of a monolayer, many layers of disinfectant can be attached to the surface. Table 5 shows that flat surfaces containing a combination of covalently and non-covalently bound disinfectants C_18_G_BAC, as well as surfaces with only non-covalently bound disinfectants (G_BAC2 and G_BAC3), demonstrate a high efficiency in inactivating the virus. For those surfaces, a complete inhibition of SARS-CoV-2 may occur even at short incubation with the virus-containing liquid (5 min). Therefore, the non-covalent binding of large amounts of BAC imparts a high anti-SARS-CoV-2 efficiency to the system.

We were interested in comparing the anti-SARS-CoV-2 activity of the non-covalently bound disinfectant with that of the free disinfectant in solution. For this aim, we prepared negatively charged silica NPs with electrostatically bound disinfectants BAC and C18-4-C18. The concentration of the disinfectants was the same in the presence and the absence of the NPs. From the concentration dependences of virucidal activity (expressed as IC values), the EC50 values were derived. As evident in Figure 7, the EC50 values for the systems with NPs are 2-4-fold higher than in the absence of NPs. The higher the disinfectant/NP ratio q, the more pronounced the effect. This means that the NPs reduce the efficiency of SARS-CoV-2 inactivation.

Thus, the non-covalent binding of a disinfectant to the surface lowers its activity against the virus. This may seem counterintuitive, since one could expect that the physical proximity of several disinfectant chains on the surface of an NP may facilitate their penetration of the viral membrane, thereby enhancing the membrane-perturbing effect responsible for virus inactivation. Nevertheless, the experimental results suggest that mostly free disinfectant molecules participate in the inactivation of the virus. This conclusion is further supported by the fact that EC50 values of many cationic surfactants active against SARS-CoV-2 are well below their critical micelle concentrations (CMCs), which means that those surfactants act in a nonaggregated state [19]. Thus, BAC has an EC50 of 0.07 mM (at 60 min of incubation time), which is much lower than its CMC (0.37 mM [19]). The idea that single surfactant ions (rather than aggregates) interact with the virus envelope membrane is consistent with the data on the interaction between cationic surfactants and phosphatidylcholine bilayers of liposomes [48].

Now let us compare the flat and structured surfaces (Table 5). In the case of the coatings with only covalently bound disinfectant, the structured surface shows a somewhat more pronounced effect (IC 10% for C_18_G_NP instead of 5.2% for flat C_18_G), which may be due to the higher surface area available for the interaction with the virus and a higher degree of NP modification in comparison with the flat surface (the disinfectant grafting density on the structured surface is 2.3-fold higher than on the flat one). In the case of coatings containing both covalently and non-covalently bound disinfectants, the effect of surface roughness is the opposite. The flat coating (C_18_G_BAC) exhibits a higher activity against SARS-CoV-2 than the structured one (C_18_G_NP_BAC): IC 100% instead of 72.6% (at 5 min of incubation time). This behavior is consistent with our hypothesis that in this system, preferentially, the surfactant ions released from the surface interact with virus particles. Indeed, one can expect that the release from the flat surface should be faster than from the crowded surface covered by NPs. But overall, the effect of surface roughness is not very pronounced.

Therefore, only the surfaces with non-covalently bound disinfectants and the surfaces combining covalently and non-covalently bound disinfectants demonstrate high activity against SARS-CoV-2 because they provide a high enough concentration of disinfectant (significantly exceeding the amount of disinfectant in a monolayer of disinfectant covalently attached to the surface).

## 3. Materials and Methods

### 3.1. Materials

The coatings were prepared on the following substrates: glass and silicon. The glass substrates were microscope slide coverslips (24 × 24 mm) from borosilicate glass (hydrolytic class I) with a thickness of 1 (0.13–0.16 mm) from Epredia (Portsmouth, NH, USA). According to the manufacturer [49], they have the following composition: silicon dioxide SiO_2_ 69–74%, sodium oxide Na_2_O 10–16%, calcium oxide CaO 5–14%, magnesium oxide MgO 0–6%, aluminum oxide Al_2_O_3_ 0–3%, and trace elements (FeO, etc.) < 5%. The silicon substrates (10 × 10 × 0.5 mm, (100) orientation) were supplied by Crystal GMBH (Berlin, Germany).

Elongated silica NPs were purchased in Nissan Chemical Corporation (Specialty Chemicals Division, Tokyo, Japan, product number 160567) in the form of 20 wt% colloidal dispersion in 2-propanol (IPA-ST-UP) and used as received. The concentration of the surface SiOH groups in the NPs was estimated with the volumetric method as 0.5 mmol/g [39]. The size of the NPs provided by the supplier was 9–15 nm (in diameter) and 40–100 nm (in length).

The surface modifier, DTSAC, in the form of 42 wt% solution in methanol (product number 435694-100ML), benzalkonium chloride BAC (>95%) containing 70% benzyldimethyldodecylammonium chloride and 30% benzyldimethyltetradecylammonium chloride (product number 12060) and 2-propanol (99.5%+, A.C.S. reagent; product number 443425-1L), all from Sigma-Aldrich (Saint Louis, MO, USA), were used as received. The surfactant C18-4-C18 was prepared by the quaternization reaction between the two-fold excess of bis(β-hydroxyethyl)oleylamine and 1,4-dibromobutane in the presence of a catalytic amount of anhydrous sodium iodide in dry acetonitrile. The synthesis is described in detail elsewhere [33]. Distilled deionized water for the preparation of the solutions was obtained using an ultrapure water purification system Milli Q (Millipore, Burlington, MA, USA).

### 3.2. Chemical Modification of the Elongated Silica Nanoparticle Surface

The chemical modification of elongated silica nanoparticles by grafting DTSAC was performed as follows [39]: 0.9 g of 20 wt% colloidal dispersion of elongated silica nanoparticles in 2-propanol (IPA-ST-UP) was mixed with a calculated amount of DTSAC ([DTSAC] = 0.4 mmol/g SiO_2_) in 17.46 g of 2-propanol while stirring at room temperature for 24 h under argon atmosphere.

### 3.3. Hydrophilization and Chemical Modification of Glass and Silicon Substrates

At first, the glass and silicon substrates were hydrophilized by treatment in a solution of acidic piranha (H_2_SO_4_:H_2_O_2_ = 5:1) for 10 min [50]. At the end of the treatment, the substrates were rinsed in deionized water and methanol. Immediately after that, chemical modification of the substrates was carried out. For this, the samples were put into 7.3 mL of methanol and then 1 mL of a 42 wt% DTSAC solution in methanol was added in an argon atmosphere. The reaction was carried out for 3 days at room temperature. Then, the samples (C_18_G and C_18_S) were immersed in distilled deionized water for 5 min and rinsed with water and methanol. The excess methanol was shaken off, and the substrates were allowed to dry at room temperature overnight.

To prepare flat surfaces combining covalently and non-covalently attached disinfectants (C_18_G_BAC sample), the glass and silicon substrates with a chemically grafted DTSAC (C_18_G and C_18_Si samples) were coated with BAC. Then, 200 µL of 0.1 wt% aqueous solution of BAC was applied to the C_18_G plates measuring 24 mm × 24 mm. The C_18_Si plates with dimensions of 8 mm × 7 mm were coated with 100 μL of 0.1 wt% aqueous solution of BAC.

To prepare structured surfaces (C_18_G_NP, C_18_Si_NP, and C_18_G_NP_BAC), the glass and silicon substrates with a chemically grafted DTSAC (C_18_G and C_18_Si samples) were coated either with modified SiO_2_ elongated NPs (C_18_G_NP and C_18_Si_NP samples) only or first with modified SiO_2_ NPs and then with BAC (C_18_G_NP_BAC sample). The coating of the DTSAC-grafted glass and silicon substrates with modified elongated SiO_2_ NPs was performed by spraying a 1 wt% NP suspension in methanol using Chemat Technology Spin-coater KW-4A in two stages (first stage at 500 rpm for 12 s; second stage at 2000 rpm for 20 s). In this way, 5 layers of NPs were made. Then, the samples were immersed in distilled deionized water for 5 min, rinsed with distilled deionized water, and dried at room temperature overnight. To form the BAC layer, 0.2 mL of 0.1 wt% BAC aqueous solution was applied to the substrates and left to dry in the open air for 3 days. The sample preparation conditions are summarized in Table 1.

### 3.4. Preparation of Glass and Silicon Substrates with Non-Covalently Attached Disinfectants

To prepare flat substrates with electrostatically bound disinfectants (G_BAC1, G_BAC2, G_BAC3, G_C18-4-C18 samples), the glass plates were kept for 24 h in an aqueous 0.1 N NaOH solution, dried in air overnight, and then a 0.1 mL of 0.019 mM, 0.19 mM, or 2.8 mM aqueous solution of BAC or 0.1 mL of a 0.0095 mM aqueous solution of C18-4-C18 were applied to the dry substrates to obtain samples G_BAC1, G_BAC2, G_BAC3, and G_C18-4-C18, respectively.

To prepare structured surfaces with electrostatically bound disinfectant (G_NP_BAC), the glass plates were kept for 24 h in an aqueous 0.1 N NaOH solution and dried in air overnight. Then, five layers of NPs were applied to the negatively charged surfaces by spin coating (4 times for 6 s at 500 rpm and 20 s at 2000 rpm) a 1 wt% colloidal dispersion of NPs in isopropanol. Then, the plates were immersed in distilled water for 5 min, rinsed with distilled water, and dried overnight in open air. After that, 200 µL of 0.1 wt% aqueous solution of BAC was applied on the dried glass plates coated with NPs and left to dry in open air overnight.

To prepare solutions of disinfectants in the presence of oppositely charged NPs (BAC/NP- and C18-4-C18/NP-, Table 3), a calculated amount of 40 wt% colloidal silica suspension in H_2_O (pH 9.2) LUDOX^®^ TM-40 (product number 420786) was dispersed in an aqueous solution of BAC or C18-4-C18, respectively, under ultrasound.

### 3.5. Measurement of Static Water Contact Angles

To assess the hydrophilicity of the glass and silicon substrates, we visually measured the contact angles *θ*^H^_2_^O^ by wetting the substrate surface with water. The accuracy of the method is 1.5°.

### 3.6. Attenuated Total Reflection Infrared (ATR-IR) Spectroscopy

IR spectra were recorded on a Bruker Vertex 70v Fourier transform IR spectrometer (Germany) by the attenuated total reflection method in the range of 4000–400 cm^−1^ with a 4 cm^−1^ resolution using a PIKE GladyATR device with diamond crystal. The details of the measurements are described elsewhere [51,52]. The measured ATR-IR spectra were corrected using OPUS 7 software, accounting for the wavelength dependence of radiation penetration depth into the sample.

### 3.7. Scanning Electron Microscopy (SEM)

Scanning electron microscopy measurements were carried out with a Hitachi SU8000 field-emission scanning electron microscope (Tokyo, Japan). Images were acquired in secondary electron mode at 24 kV accelerating voltage. The target-oriented approach was used to optimize the measurements [53]. The samples were mounted on a 25 mm aluminum specimen stub, fixed by conductive carbon tape, and coated with a 20 nm film of carbon.

### 3.8. X-Ray Photoelectron Spectroscopy (XPS)

X-ray photoelectron spectra were acquired with an Axis Ultra DLD (Kratos, Manchester, UK) spectrometer using monochromatized Al Kα (1486.6 eV) radiation at an operating power of 150 W for the X-ray tube. Survey and high-resolution spectra of appropriate core levels were recorded at pass energies of 160 and 40 eV and with step sizes of 1 and 0.1 eV, respectively. A sample area of 300 μm × 700 μm contributed to the spectra. The samples were mounted on a sample holder with two-sided adhesive tape, and the spectra were collected at room temperature. The base pressure in the analytical UHV chamber of the spectrometer during measurements did not exceed 10^−8^ Torr. The energy scale of the spectrometer was calibrated to provide the following values for reference samples (i.e., metal surfaces freshly cleaned by ion bombardment): Au 4f_7/2_–83.96 eV, Cu 2p_3/2_–932.62 eV, Ag 3d_5/2_–368.21 eV. The electrostatic charging effects were compensated for by using an electron neutralizer. Sample charging was corrected by referencing the C-C/C-H peak identified in the C ls spectrum (284.8 eV) related to adventitious carbon or modifier. After charge referencing, a Shirley-type background with inelastic losses was subtracted from the high-resolution spectra. The surface chemical composition was calculated using atomic sensitivity factors included in the software of the spectrometer, corrected for the transfer function of the instrument.

### 3.9. Cells

Vero E6 (ATCC, Manassas, VA, USA; catalog number CRL-1586), a cell line with epithelial morphology derived from the kidney of an African green monkey (*Chlorocebus* sp.), is widely used for growing slow-replicating viruses, including SARS-CoV-2, to which it is highly susceptible [54,55]. The cells were cultured at 37 °C and 5% CO_2_ in Dulbecco’s modified Eagle’s medium that contained 4.5 g/L α-D-glucose (DMEM; Sigma-Aldrich, St. Louis, MO, USA) and was supplemented with 5% fetal calf serum (FCS), 2 mM L-glutamine, and 150 u/mL penicillin–streptomycin (growth medium). All supplements were from Thermo Fisher Scientific (Waltham, MA, USA).

### 3.10. Virus and Virus Titration

The SARS-CoV-2 isolate was from the collection of viruses of the Gamaleya National Research Center for Epidemiology and Microbiology (hCoV-19/Russia/Moscow-PMVL-12/2020; lineage B.1.1.4 [56]; GISAID reference EPI_ISL_572398). The viral stock was grown in confluent Vero E6 monolayers for four days, pelleted by centrifugation (140,000× *g*, 4 °C, 1 h; Optima XPN 100, Beckman Coulter, Brea, CA, USA), resuspended at 1 × 10^8^ median tissue culture infectious doses (TCID_50_) per 1 mL, and stored in aliquots at −80 °C.

One TCID_50_ is the amount of a virus that irreversibly alters cell morphology in 50% of inoculated cultures; the morphological alterations thus produced are termed cytopathic effects (CPEs). Measuring TCID_50_ by endpoint dilution is a standard method of SARS-CoV-2 quantification [57]; 10 TCID_50_ was shown to be equivalent to 2–4 infectious virions [58,59] (which is two to three times lower than the theoretical estimation [60]).

Vero E6 cells (1.2 × 10^6^ cells/mL growth medium) were seeded into 96-well flat-bottomed Costar tissue culture plates (Corning, Corning, NY, USA) at 100 µL/well and incubated at 37 °C and 5% CO_2_ for 24 h. The resulting confluent monolayers were washed with FCS-free DMEM (2 × 5 min). Serial 10-fold dilutions of the virus in support medium (DMEM, 1% FCS) were then introduced at 100 µL/well to give final titers in the range of 10^1^–10^8^ TCID_50_/mL. Following incubation at 37 °C and 5% CO_2_ for 2 h, during which the virus adsorption was completed, the medium with the inoculum was removed. The monolayers were washed with FCS-free DMEM (2 × 5 min), 100 µL support medium (DMEM, 2% FCS) was added to each well, and the plates were further incubated at 37 °C and 5% CO_2_ for 96 h. The cultures were examined once daily to follow the development of virus-induced CPEs. For each virus dilution, TCID_50_ measurement was performed in octuplicates, and eight wells in every plate served as a virus-free control.

To complement visual examination, cell viability was determined using the CellTiter 96^®^ AQueous One Solution Cell Proliferation Assay (Promega, Madison, WI, USA; catalog number G3582). This test is based on the ability of live cells to convert 3-(4,5-dimethylthiazol-2-yl)-5-(3-carboxymethoxyphenyl)-2-(4-sulfophenyl)-2H-tetrazolium inner salt (MTS) into a water-soluble colored formazan (the conversion, effected by dehydrogenase-catalyzed reaction-derived NAD(P)H [61,62], is proportional to the concentration of viable cells). At the end of the 96 h incubation, the culture medium was withdrawn, and 100 µL of support medium (DMEM, 2% FCS) and 20 µL MTS reagent were added to each well, followed by an additional incubation at 37 °C for 3 h. The optical density was measured at 490 nm on an iMark plate reader (Bio-Rad Laboratories, Hercules, CA, USA) using 630 nm as a reference wavelength.

The percentages of cultures with CPEs and viability loss were calculated for each virus dilution (the results obtained using both methods were in good agreement with each other). Virus titers were calculated by a modification of the Reed–Muench method [63,64] and expressed in TCID_50_/0.1 mL or lg TCID_50_/0.1 mL.

### 3.11. Assessment of Activity Against SARS-CoV-2

The capacity for replication is the sole criterion of the viability of a virus [65,66]. This is the reason why the virucidal activity of the coatings under study was assessed by comparing the replication competence of SARS-CoV-2 virions exposed to surfactant-modified and surfactant-free surfaces.

A 100 µL aliquot of a suspension of the virions (10^8^ TCID_50_/mL) was spread over the surface (surfactant-modified or surfactant-free) of the glass or silicon plates and left at ambient temperature for 5 min or 60 min. The suspensions from each surface were collected, and the virions were pelleted by centrifugation (140,000× *g*, 1 h; Optima XPN 100, Beckman Coulter, Brea, CA, USA) and resuspended in 300 µL of support medium (DMEM, 1% FCS) to rule out adverse effects of surfactants on the cells. For each pellet, the titer of infectious SARS-CoV-2 was determined as described in Section 3.10 above (the only difference being that TCID_50_ measurement for each dilution was performed in triplicates). Thus, replication-competent SARS-CoV-2 virions were quantified directly (by measuring the extent to which the infection they induced was pronounced).

The virucidal effects of surfactant-modified surfaces were assessed by the difference in the virus titers (A) between the control (A_c_) and experimental (A_e_) samples (i.e., virion suspensions exposed, respectively, to surfactant-free and surfactant-modified surfaces) as follows:

A = A_c_ − A_e_

The formula

IC = [(A_c_ − A_e_)/A_c_] × 100%

was used to calculate the inhibition coefficient (IC) for all variants of the experiment (surface material, surfactant type, surfactant concentration, exposure time, etc.).

Concentration–response curves were obtained by plotting IC values against surfactant concentrations, and the values of EC50 were determined by non-linear regression analysis (Prism 6.01; GraphPad Software, San Diego, CA, USA). The 4-parametric equation of the logistic curve was taken as a working model for EC50 analysis (menu items “Nonlinear regression”—“Sigmoidal dose-response (variable slope)”). For the analysis of the IC50, a 4-parametric equation of the logistic curve was used (menu items “Nonlinear regression”—“log (inhibitor) vs. response (variable slope)”).

### 3.12. Cytotoxicity Tests

Serial dilutions of the surfactants in support medium (DMEM, 2% FCS) were added to confluent Vero E6 monolayers in 96-well plates, and the plates were incubated at 37 °C in 5% CO_2_ for 72 h. Each dilution was tested in quadruplicates; eight wells served as a vehicle control. Cell viability was assessed by the MTS test (CellTiter 96^®^ AQueous One Solution Cell Proliferation Assay; Promega, Madison, WI, USA) based on the ability of live cells to convert 3-(4,5-dimethylthiazol-2-yl)-5-(3-carboxymethoxyphenyl)-2-(4-sulfophenyl)-2H-tetrazolium inner salt (MTS) into a colored formazan product that is soluble in tissue culture medium [61,62]. When the incubation was completed, the culture medium was removed from the wells, and 100 µL of support medium (DMEM, 2% FCS) and 20 µL MTS reagent were added to each well, followed by plate incubation at 37 °C for an additional 3 h. Absorbance was measured at 490 nm on an iMark plate reader (Bio-Rad Laboratories, Hercules, CA, USA), using 630 nm as a reference wavelength. The values of CC50 were derived from viability dependences on surfactant concentrations using non-linear regression analysis (Prism 6; GraphPad Software, San Diego, CA, USA).

## 4. Conclusions

The recent COVID-19 pandemic has led to a surge in demand for virus inactivation technologies. The use of chemical disinfectants has long been a widely accepted practice for infection prevention and control [67]. In the present paper, three types of quaternary ammonium disinfectant coatings inactivating SARS-CoV-2 were investigated and compared as follows: (i) coatings with covalently bound disinfectant, (ii) coatings with non-covalently bound disinfectant, and (iii) coatings with both covalently and non-covalently bound disinfectants. The coatings with covalently bound disinfectant appear to be more attractive at first glance, as the disinfectant does not leach out over time. However, our research shows that these coatings have relatively low activity against SARS-CoV-2 for the following reasons: the small amount of disinfectant in the monolayer attached to the surface and the reduced efficacy of the bound disinfectant compared with the free one. The coatings with non-covalently bound disinfectants and coatings combining covalently and non-covalently bound disinfectants are much more promising. They demonstrate high activity against SARS-CoV-2 in the suspension test [68] with complete inhibition of the virus occurring even after a short incubation period with virus-containing liquid (5 min). Although the activity of these coatings will decrease over time because of the release of the disinfectant, it can be easily restored by applying a new layer of the disinfectant (e.g., by spraying).

Also, in the present study, a recently synthesized new gemini quaternary ammonium surfactant C18-4-C18 was tested for activity against SARS-CoV-2. Its EC50 is found to be 0.03 mM, which is 2.3-fold lower than that of one of the most widely used quaternary ammonium disinfectants—BAC. It can be further used in the formulations of effective disinfectants for hands and surfaces, which may help to limit the spread of infections.

## Figures and Tables

**Table 1:** The surface coatings under study.

Sample	Substrate Material	Type of Surfactant Binding to the Surface *	Type of Surface	Stages of Surface Modification		
				Grafting DTSAC	Application of Modified SiO_2_ NPs by Spin-Coating	Application of BAC
G_BAC	glass	noncov	flat	-	-	+
C_18_G	glass	cov	flat	+	-	-
C_18_G_BAC	glass	cov + noncov	flat	+	-	+
C_18_G_NP	glass	cov	structured	+	+	-
C_18_G_NP_BAC	glass	cov + noncov	structured	+	+	+
C_18_Si	Si	cov	flat	+	-	-
C_18_Si_NP	Si	cov	structured	+	+	-

* cov—covalent, noncov—non-covalent.

**Table 2:** Atomic composition of the surface of the samples under study.

Sample	Elements, %					Method
	Si	C	N	O	H	
C_18_G	17.2	42.9	1.3	37.8		XPS
C_18_Si	18.9	58.5	1.1	21.5		XPS
NPs		7.54	0.28		1.98	Elemental analysis

**Table 3:** Characteristics of the N 1s XPS spectra: binding energy E_b_, Gaussian width W, and relative intensity I_rel_ of photoelectron peaks.

Sample	Parameters	Peak 1	Peak 2	Peak 3
C_18_G	E_b_, eV	402.5	399.7	-
	W, eV	1.77	1.93	-
	I_rel_	0.6	0.4	-
C_18_Si	E_b_, eV	402.3	399.9	398.7
	W, eV	1.1	1.37	1.64
	I_rel_	0.41	0.47	0.12

**Table 4:** Cytotoxicity of cationic surfactants to Vero E6 cells.

Sample	CC50, mM
BAC	0.3
DTSAC	4.2
C18-4-C18	0.8
C18-4-C18 with added NPs *	3.4

* [C18-4-C18]/[SiO_2_] = 0.6.

**Table 5:** SARS-CoV-2 inactivation on glass G and silicon Si platelets.

Sample	Disinfectant Grafting Density, Group/nm^2^	Contact Time, min	Virus Titer			Inhibition Coefficient IC, %
			Control A_c_	Experiment A_e_	Log10 Reduction A	
Flat surfaces						
C_18_G	-	60	7.3	6.9	0.4	5.2
C_18_G_BAC	290	5	7.3	0.0	7.3	100
		60	7.0	0.0	7.0	100
G_BAC1	1	60	7.0	7.0	0.0	0
G_BAC2	10	60	7.0	0.0	7.0	100
G_BAC3	290	5	8.3	0.0	8.3	100
		60	7.0	0.0	7.0	100
G_C18-4-C18	1	60	7.0	4.7	2.3	32.9
C_18_Si	1	60	6.0	4.75	1.25	20.8
Unmodified G	0	60	7.7	7.7	0.0	0
Structured surfaces						
C_18_G_NP	2.3	60	7.0	6.3	0.7	10.0
C_18_G_NP_BAC	290	5	7.3	2.0	5.3	72.6
		60	7.0	0.0	7.0	100
G_NP_BAC	290	5	8.3	0.0	8.3	100
C_18_Si_NP	2.3	60	6.0	4.75	1.25	20.8

## Data Availability

Data are contained within the article.
